# Nivolumab dose selection: challenges, opportunities, and lessons learned for cancer immunotherapy

**DOI:** 10.1186/s40425-016-0177-2

**Published:** 2016-11-15

**Authors:** Shruti Agrawal, Yan Feng, Amit Roy, Georgia Kollia, Brian Lestini

**Affiliations:** 1 Clinical Pharmacology and Pharmacometrics, Exploratory Clinical and Translational Research, Bristol-Myers Squibb Co., 3551 Lawrenceville Road, Princeton, NJ 08543 USA; 2 Global Biometric Sciences, Bristol-Myers Squibb Co., Princeton, NJ 08543 USA; 3 Global Clinical Research, Bristol-Myers Squibb Co., Princeton, NJ 08543 USA

**Keywords:** Dose selection, Immunotherapy, Melanoma, Nivolumab, Non-small cell lung cancer, Renal cell carcinoma

## Abstract

**Background:**

Immuno-oncology (I-O) therapies target the host immune system, providing the potential to choose a uniform dose and schedule across tumor types. However, dose selection for I-O agents usually occurs early in clinical development and is typically based on tumor response, which may not fully represent the potential for improved overall survival. Here, we describe an integrated approach which incorporates clinical safety and efficacy data with data obtained from analyses of dose-/exposure-response (D-R/E-R) relationships, used to select a monotherapy dose for nivolumab, a programmed death–1 inhibitor, in clinical studies of different tumor types.

**Methods:**

Dose was selected based on anti-tumor activity and safety data from a large phase 1b, open-label, dose-escalation study of nivolumab at doses ranging from 0.1 to 10 mg/kg administered every 2 weeks (Q2W) in 306 patients with advanced malignancies, and quantitative analyses were performed to characterize D-R/E-R relationships for pharmacodynamic, safety, and efficacy endpoints.

**Results:**

A maximum tolerated dose for nivolumab was not identified, and the safety profile was similar across tumor types and dose levels (0.1–10 mg/kg). Objective response rates (ORRs) were similar across doses in melanoma and renal cell carcinoma (RCC), while higher ORRs were observed in non-small cell lung cancer (NSCLC) at 3 mg/kg and 10 mg/kg versus 1 mg/kg. Peripheral receptor occupancy was saturated at doses ≥ 0.3 mg/kg. In D-R/E-R analyses, a positive dose-dependent objective response trend was observed for each tumor type, but appeared to plateau at nivolumab doses of ≥ 1 mg/kg for melanoma and RCC, and at ≥ 3 mg/kg for NSCLC. Although there was no apparent relationship between tumor shrinkage rate and exposure, tumor progression rate appeared to decrease with increasing exposure up to a dose of 3 mg/kg Q2W for NSCLC.

**Conclusions:**

Nivolumab monotherapy at 3 mg/kg Q2W provides unified dosing across tumor types. This dose and schedule has been validated in several phase II/III studies in which overall survival was an endpoint. Integrating D-R/E-R relationships with efficacy data and a safety profile that is unique to I-O therapy is a rational approach for dose selection of these agents.

**Electronic supplementary material:**

The online version of this article (doi:10.1186/s40425-016-0177-2) contains supplementary material, which is available to authorized users.

## Background

The oncology treatment landscape has changed considerably in the past decade due to a deeper understanding of tumor biology and tumor-immune interactions at a molecular level. Although there have been clear advances, room remains for significant improvement with regard to the success rate of pivotal clinical trials [1]. A possible reason for this shortfall is that the dose selected to be tested in pivotal trials has traditionally been based on the maximum tolerated dose (MTD) paradigm. Although often appropriate for cytotoxic drugs, the MTD may not be the best approach for selecting phase III doses of targeted and immuno-oncology (I-O) agents, especially for well-tolerated agents, where MTD may not even be determined [2]. Furthermore, the toxicity profile of targeted and I-O agents differs from that of cytotoxic therapy, and the typical 4-week observation period that is used to identify dose-limiting toxicities (DLTs) may not be sufficient. I-O treatments are associated with a distinctive class of immune-related adverse events (AEs) for which exposure-safety relationships are not yet well understood. Moreover, in early-stage clinical trials when exposure-response (E-R) relationships are typically analyzed, data are often limited by low numbers of patients and limited exposure durations. Taken together, these factors can contribute to selecting a suboptimal biological dose [3].

Dose selection for I-O treatments presents additional unique challenges. Overall survival (OS) is considered the standard endpoint for anti-cancer agents. However, OS is associated with long follow-up times and other early clinical efficacy endpoints (such as Response Evaluation Criteria In Solid Tumors [RECIST] tumor response) may not fully represent the potential clinical benefit in pivotal trials [4]. In vivo models are often limited in predicting clinical efficacy of a given dose and schedule of an I-O agent. Taken together, these factors make phase III dose and schedule selection challenging for I-O agents.

Conversely, I-O drugs may allow identifying a uniform monotherapy dose and scheduling across tumor types and stages of disease. This hypothesis is based on the fact that their mechanisms of action promote anti-tumor activity through direct effects on immune regulatory pathways. Thus, while the characteristics of different tumor types may vary widely, anti-tumor immune response is a core mechanistic feature of I-O agents. Several I-O agents have been approved, including ipilimumab, a cytotoxic T-lymphocyte antigen–4 (CTLA-4) immune checkpoint inhibitor, as well as the programmed death–1 (PD-1) immune checkpoint inhibitors nivolumab and pembrolizumab and the PD-1 ligand (PD-L1) inhibitor atezolizumab. However, one of the earliest, if not the first, published investigations of E-R relationships for an I-O agent was performed with ipilimumab [5, 6]. In those analyses, which were based on population pharmacokinetics (PPK) modeling, higher steady-state trough concentrations (Cminss) were associated with improved OS and increased anti-tumor activity but with a higher incidence of immune-related AEs. Ultimately, ipilimumab monotherapy demonstrated superior OS in a phase III trial in patients with previously treated metastatic melanoma at a dose level and schedule of 3 mg/kg every 3 weeks (Q3W) for up to four doses [7]. These results indicate that characterizing the relationships between pharmacokinetics (PK) (ie, exposure) and key clinical outcomes from phase I and II trials represents a valid approach to dose selection for I-O agents.

Here, we describe the multifactorial considerations employed in selecting a monotherapy dosing regimen that was investigated in phase III trials of nivolumab across three tumor types (melanoma, non-small cell lung cancer [NSCLC], and renal cell cancer [RCC]). The phase III dosing regimen was selected based on an integrated analysis of safety and efficacy across tumor type and dose level, and dose-response (D-R)/E-R relationships of efficacy, safety, and pharmacodynamic biomarkers from a large phase Ib study in patients with advanced or recurrent solid malignancies [8]. The nivolumab dosing regimen selected was shown to be safe and effective in four large, randomized, controlled trials [9–12].

## Methods

### Patients and study design

Patients included in the nivolumab dose selection analysis (*N* = 306) had participated in a large phase Ib open-label, dose-escalation, cohort-expansion study to evaluate the anti-tumor activity and safety of nivolumab in previously treated advanced or recurrent malignancies across advanced melanoma, NSCLC (including both squamous and non-squamous), RCC, metastatic castration-resistant prostate cancer (mCRPC), or colorectal cancer (CRC) (CA209-003; Additional file 1: Figure S1) [8]. Patients received nivolumab at doses ranging from 0.1 to 10 mg/kg every 2 weeks (Q2W) administered as an intravenous infusion, for up to 2 years, unless they had a complete response (CR), unacceptable AEs, progressive disease (PD), or withdrew consent. Tumor response assessments were conducted every 8 weeks. All participants, or their legal representatives, gave written informed consent prior to enrollment.

Patients across tumor types were enrolled into cohorts of 3–6 per dose level. Dose escalation proceeded when a minimum of three patients had completed the safety evaluation period (56 days) at a given dose level, with DLTs observed in less than one third of patients. Intra-patient dose escalation was not permitted.

Further dose-expansion cohorts were enrolled for melanoma (assigned to a dose of 0.1, 0.3, 1, 3, or 10 mg/kg), NSCLC (either squamous or non-squamous, assigned to a dose of 1, 3, or 10 mg/kg), and RCC (at a dose of 1 or 10 mg/kg).

### Safety

Safety evaluations were conducted for all treated patients at baseline and at regular intervals. The severity of AEs was graded according to the National Cancer Institute Common Terminology Criteria for Adverse Events, v3.0 [13]. AEs were coded with the use of the Medical Dictionary for Regulatory Activities (MedDRA), version 15.1. Select AEs, defined as those with a potential immunologic cause, were coded with the use of a predefined list of MedDRA terms.

### Efficacy

The efficacy population consisted of patients in whom the response could be evaluated and who had measurable disease at baseline with one of the following: at least one scan obtained during treatment, clinical evidence of disease progression, or death.

Efficacy endpoints included objective response rate (ORR) and progression-free survival at 24 weeks (PFSR 24). ORR was based on best overall response (BOR) of CR and partial response (PR) as derived by the sponsor using RECIST v1.1 criteria [14]. Objective responses were confirmed by at least one sequential tumor assessment, scheduled at screening and between days 52 and 56 of each cycle, and ORR was calculated as (CR + PR)/number of patients × 100 in the study population. The confidence intervals for ORR were based on the Clopper Pearson interval.

PFSR 24 was calculated as the proportion of patients without disease progression or death at 24 weeks, according to the Kaplan-Meier method, with confidence intervals using the Greenwood method.

### Integrated PK, D-R/E-R analyses

#### Nivolumab PK

Nivolumab PK was characterized by an integrated PPK approach with intensive and sparse PK data from 343 patients with solid tumors, who were enrolled in a pilot phase I study (*N* = 39) and large phase Ib (*N* = 304) study [8, 15]. The effects of the following covariate-parameter relationships were estimated in the PPK model: baseline body weight, age, sex, estimated glomerular filtration rate (GFR), lactate dehydrogenase (LDH), albumin, total bilirubin, C-reactive protein, and absolute lymphocyte count on clearance and body weight and sex on central volume of distribution. Visual predictive check was used to evaluate the performance of the PPK model, and summary measures of steady-state trough, peak, and time-averaged concentration (Cminss, Cmaxss, and Cavgss) were determined for each patient for whom nivolumab concentration data were available [16].

#### Pharmacodynamics

To determine D-R of the pharmacodynamic biomarker, receptor occupancy (RO), serum concentrations of nivolumab were quantified with the use of an enzyme-linked immunosorbent assay. In the phase Ib study, peripheral-blood mononuclear cells were isolated from patients at baseline and after the first treatment cycle to estimate RO of PD-1 by nivolumab on circulating CD3+ T cells by means of flow cytometry at baseline and week 8 post-treatment [8, 15]. The available RO data were fitted to a maximum possible effect (Emax) model as a function of nivolumab concentration [15].

#### D-R analyses for safety and tolerability

D-R relationships of safety and tolerability were examined with respect to grade ≥ 3 AEs, AEs leading to discontinuation, as well as maintenance of dose-intensity. The D-R of time-to-event of grade ≥ 3 AEs and AEs leading to discontinuation were described by Kaplan-Meier analyses of pooled safety data across tumor types of patients enrolled in the phase Ib study. Dose interruption was allowed in the phase Ib study, and tolerability was also examined graphically by plotting dose intensity versus time.

#### E-R analyses for efficacy

Nivolumab E-R relationships of efficacy were investigated with respect to confirmed objective response, and tumor growth dynamics (TGD). Assessment of the relationship of nivolumab Cminss and TGD model estimated shrinkage and progression rates complements the more conventional assessment of efficacy by OR, by enabling an assessment of the nivolumab on the entire longitudinal time-profile of tumor response that goes beyond the effect on categories of BOR.

The E-R of OR was described by separate logistic regression models for each of the following tumor types: melanoma, NSCLC, and RCC. Linear, log-linear, and non-parametric (restricted cubic spline) functional forms of Cminss were assessed in the logit function. Potentially modulatory covariate effects were not included in E-R models, given the limited data available at the time of dose selection.

**Fig. 1:**
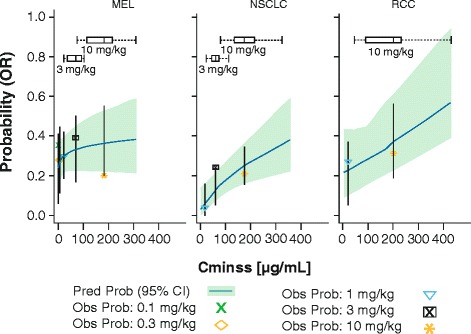
Exposure-response efficacy analysis of nivolumab by tumor type. Vertical lines represent 90 % prediction intervals for each dose level. CI = confidence interval; Cminss = steady-state nivolumab trough concentration; MEL = melanoma; NSCLC = non-small cell lung cancer; Obs Prob = observed probability; OR = objective response; Pred Prob = predicted probability; RCC = renal cell carcinoma

The logistic regression analysis of E-R of OR found that the probability of OR was best described by log-transformed steady-state trough concentration (Cminss), in each of the tumor types (melanoma, NSCLC, and RCC), which suggests that the probability of OR increases with increasing Cminss for each of these tumor types (Fig. 1). However, the observed proportion of responders was highest with 3 mg/kg in each of these tumor types. Exploratory analysis of OR revealed that the subset of patients with higher Cminss within a dose tended to respond better than some patients at higher doses who had even higher Cminss. Thus, further exploratory analysis was performed to investigate the reason for the inconsistency between the observed and model predicted responses.

The time course of tumor size was characterized by a non-linear mixed-effect TGD model described previously [17]. Specifically, the tumor size (sum of longest diameter of index lesions) at each tumor assessment visit was described by the following equation:

$$ T{S}_i(t)=BT{S}_{(i)}\times {e}^{-S{R}_it}+P{R}_i\times t, $$

where *TS*_*i*_(*t*) is the tumor size at time *t* for the *i*th patient; and *BTS*_*i*_, *SR*_*i*_, and *PR*_*i*_ represent baseline tumor size, tumor shrinkage rate constant, and linear tumor progression rate for the *i*th patient, respectively.

The relationship between nivolumab Cminss and TGD model parameters (*SR* and *PR*) were evaluated by tumor type.

## Results

A total of 306 patients with advanced solid tumors, including melanoma (*n* = 107), NSCLC (*n* = 129 [including 74 with non-squamous, 54 with squamous, and 1 with unknown histology]), RCC (*n* = 34), CRC (*n* = 19), and mCRPC (*n* = 17) received treatment with nivolumab monotherapy in the phase Ib study between October 2008 and March 2013 (see Additional file 2: Table S1). Baseline characteristics have been described previously [8]. Safety data are presented for all patients who received at least one dose of nivolumab. Efficacy data are presented for 270 patients with melanoma, NSCLC, and RCC. The protocol specified dosing frequency was Q2W for all patients in the study.

No MTD was identified up to the highest dose tested (10 mg/kg Q2W). Overall, nivolumab was considered safe and tolerable up to 10 mg/kg Q2W. The median duration of therapy across all tumor types and doses was 16.1 weeks (Additional file 3: Table S2 and Additional file 4: Figure S2). A relative dose intensity of ≥ 90 % was achieved in 265 (86.6 %) treated patients. Based on dose intensity, patients received 10 mg/kg Q2W without continued discontinuations.

**Fig. 2:**
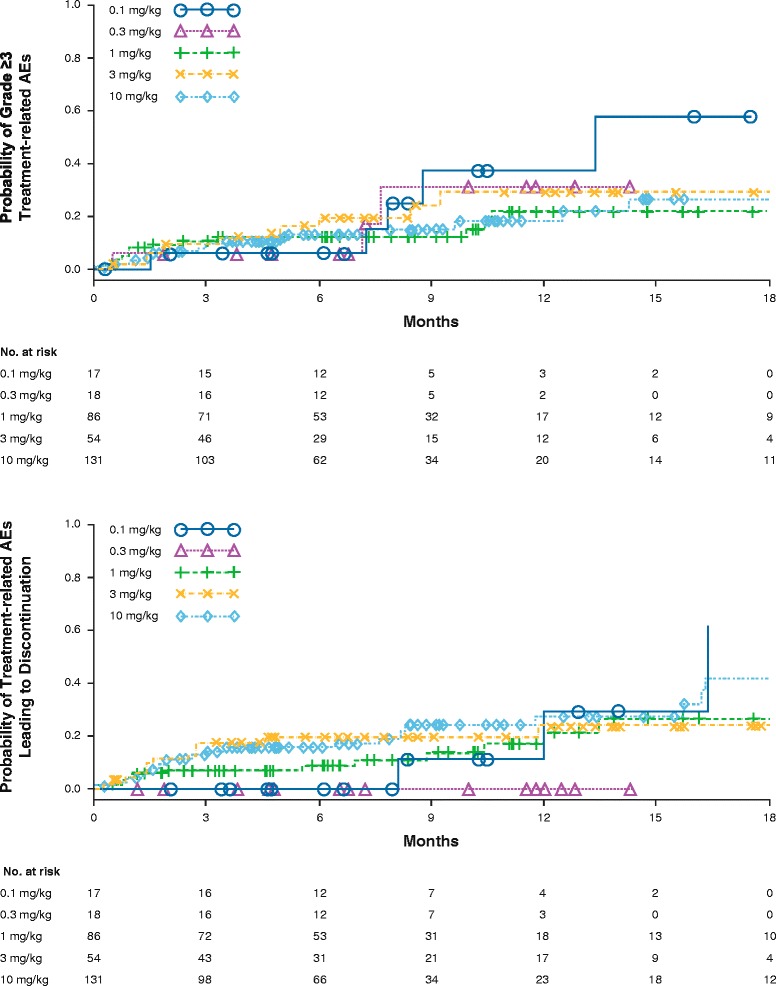
Integrated dose-response for treatment-related grade ≥3 AEs and AEs leading to discontinuation. AEs = adverse events

Overall, the safety profile of nivolumab monotherapy was generally manageable and was consistent with the mechanism of action of nivolumab. No MTD was reached at doses tested up to 10 mg/kg Q2W. The nature, frequency, and severity of treatment-related AEs were similar across dose levels (Table 1) and tumor types (Table 1 and Fig. 2), as were AEs leading to discontinuation. The most common reason for discontinuation was disease progression (*n* = 193, 67.5 %). Of all treated patients, 43 (14.1 %) delayed study drug and 11 (3.8 %) discontinued permanently due to an AE. Deaths were reported in 75 patients (24.5 %) within 100 days of the last dose of nivolumab. While most deaths (70 of 75; 93 %) were due to malignant disease, a total of five deaths were due to treatment-related pneumonitis (four with NSCLC and one with CRC) at doses of 1 (*n* = 2), 3 (*n* = 2), and 10 mg/kg (*n* = 1), which occurred independent of dose. Generally, AEs were manageable and reversible with the use of immuno-suppressants.

**Table 1:** Safety profile of nivolumab by dose level and tumor type

	Dose (mg/kg Q2W)					
Patients with treatment-related AEs, % (n)	0.1	0.3	1	3	10	Total
	(*N* = 17)	(*N* = 18)	(*N* = 86)	(*N* = 54)	(*N* = 131)	(*N* = 306)
Any grade	77 (13)	78 (14)	81 (70)	74 (40)	71 (93)	75 (230)
Grade 3/4	29 (5)	17 (3)	14 (12)	20 (11)	16 (21)	17 (52)
Serious grade 3/4	6 (1)	0	5 (4)	9 (5)	11 (14)	8 (24)
Leading to DC	18 (3)	0	11 (9)	7 (4)	12 (16)	11 (32)
Deaths	–	–	2 (2)	4 (2)	1 (1)	2 (5)
	Tumor type					
Patients with treatment-related AEs, % (n)	NSCLC	MEL	RCC	CRC	mCRPC	Total
	(*N* = 129)	(*N* = 107)	(*N* = 34)	(*N* = 19)	(*N* = 17)	(*N* = 306)
Dose levels (mg/kg)	1–10	0.1–10	1, 10	10	10	
Any grade	71 (91)	84 (90)	85 (29)	58 (11)	53 (9)	75 (230)
Grade 3/4	14 (18)	22 (24)	18 (6)	16 (3)	6 (1)	17 (52)
Serious grade 3/4	6 (8)	9 (10)	9 (3)	11 (2)	6 (1)	8 (24)
Leading to DC	12 (16)	9 (10)	9 (3)	16 (3)	0	11 (32)
Deaths	3 (4)	–	–	5 (1)	–	2 (5)

*AE* adverse event, *CRC* colorectal cancer, *DC* discontinuation, *mCRPC* metastatic castration-resistant prostate cancer, *MEL* melanoma, *NSCLC* non-small cell lung cancer, *Q2W* every 2 weeks

There was no apparent relationship between the incidence of select AEs and the dose of nivolumab. Integrated D-R for safety was assessed with respect to the cumulative time-to-event distribution of grade ≥ 3 treatment-related AEs and AEs leading to discontinuation across all tumor types in the dose-ranging phase Ib study (Fig. 2). The probability of AEs leading to discontinuation appeared to be lower in doses ≤ 1 mg/kg compared with the 3 and 10 mg/kg doses. The probabilities of both grade ≥ 3 treatment-related AEs and those leading to discontinuation were similar between 3 and 10 mg/kg doses. In addition, relative dose intensity across dose levels appeared to be > 90 % for all dose levels. The average dose intensity per patient was 1.0, 2.9, and 9.8 mg/kg/2 weeks for 1.0, 3.0, and 10.0 mg/kg dose levels, respectively.

Overall, with no established MTD, similar dose intensity, nature, and frequency of AEs across dose levels, and manageable safety profile of nivolumab, 10 mg/kg Q2W was considered safe and tolerable.

**Fig. 3:**
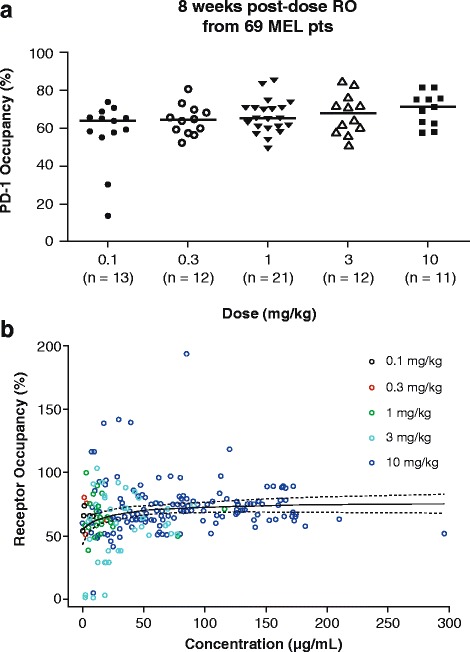
Peripheral **a** PD-1 occupancy and **b** receptor occupancy (RO) of patients treated with nivolumab. MEL = melanoma; PD-1 = programmed death–1; pts = patients

In the 69 evaluated patients with melanoma, peripheral PD-1 RO was saturated at ≥ 0.3 mg/kg doses after 8 weeks (Fig. 3a). The E-R relationship for RO is shown in Fig. 3b. Peripheral RO is saturated at a lower concentration with doses corresponding to ≥ 0.3 mg/kg. In addition, there were minor increases in activated T cells in peripheral blood, with no evidence of D-R. The peripheral pharmacodynamics data did not differentiate activity by dose level. However, it should be noted that the relationship between peripheral and intra-tumoral PD-1 RO and T-cell proliferation has not been established and may have limited value in understanding D-R relationships.

ORRs were similar across the evaluated dose ranges for melanoma and RCC. However, higher ORRs were observed for NSCLC at 3 (24.3 %) and 10 mg/kg (20.3 %) than at 1 mg/kg Q2W (3 %) (Table 2). The PFSR 24 was numerically higher at 3 mg/kg for melanoma and NSCLC than at other doses tested (melanoma: 0.1, 0.3, 1, and 10 mg/kg; NSCLC: 1 and 10 mg/kg). For RCC, ORRs were similar for 1 and 10 mg/kg, and PFSR 24 was numerically higher at 10 mg/kg than at 1 mg/kg (Table 2).

**Table 2:** ORR and PFSR 24 rates by dose in patients with melanoma, NSCLC, and RCC treated with nivolumab monotherapy

	MEL (*N* = 107)		NSCLC (*N* = 129)		RCC (*N* = 34)	
Dose (mg/kg)	ORR % (n/N)	PFSR 24, %	ORR % (n/N)	PFSR 24, %	ORR % (n/N)	PFSR 24, %
0.1	35 (6/17)	41	—	—	—	—
0.3	28 (5/18)	35	—	—	—	—
1	31 (11/35)	51	3 (1/33)	26	28 (5/18)	50
3	41 (7/17)	55	24 (9/37)	40	—	—
10	20 (4/20)	35	20 (12/59)	33	31 (5/16)	67

— = not tested, *MEL* melanoma, *NSCLC* non-small cell lung cancer, *ORR* objective response rate, *PFSR 24* progression-free survival rate at 24 weeks, *RCC* renal cell carcinoma

The E-R for efficacy was evaluated for multiple endpoints, such as objective response (OR), representing early clinical activity and TGD modeling, which is independent of follow-up and not affected by unconventional responses seen with cancer immunotherapy. In order to characterize the E-R for efficacy with OR, a log-linear function of Cminss was selected for logistic regression for three tumor types. As shown in Fig. 1, a trend was observed between the probability of OR and higher Cminss, but this appeared to plateau at doses ≥ 1 mg/kg for melanoma and ≥ 3 mg/kg for NSCLC. The E-R relationship for RCC appeared linear, although it should be noted that only two doses (1 and 10 mg/kg) were evaluated. However, given the confidence intervals, there appeared to be no difference in response rates at these two doses.

**Fig. 4:**
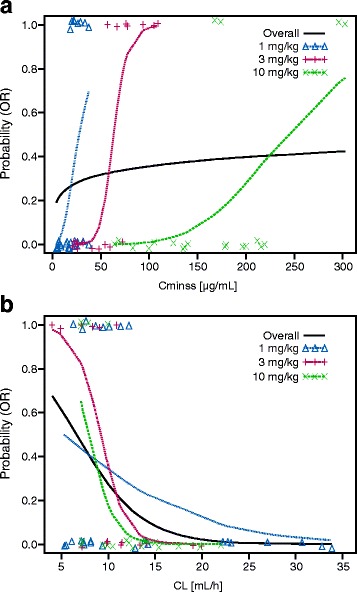
Exposure-response for efficacy by dose level in melanoma. **a** Probability of OR vs Cminss, overall and by dose. **b** Probability of OR vs CL, overall and by dose. CL = clearance; Cminss = steady-state trough concentration; OR = objective response

**Fig. 5:**
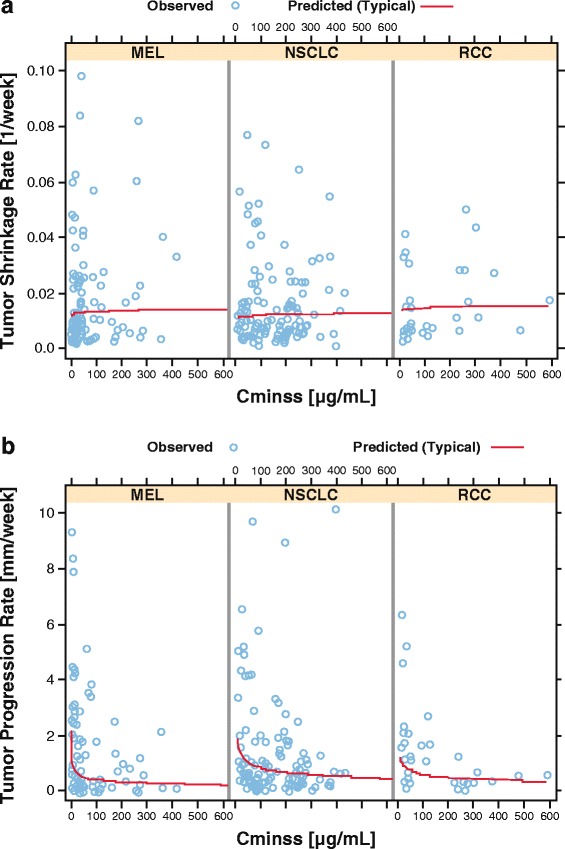
Relationship between **a** exposure and tumor shrinkage rate, and **b** exposure and tumor progression rate. Cminss = steady-state trough concentration; MEL = melanoma; NSCLC = non-small cell lung cancer; RCC = renal cell carcinoma

Exploratory analyses revealed that the responders at each dose level tended to be clustered at the higher exposures compared with non-responders at the same dose level. Additionally, some of the patients tended to respond better than others at higher dose levels who had higher exposures. This phenomenon was further evaluated by performing E-R analysis by dose (Fig. 4 for melanoma and Additional file 5: Figure S3 and Additional file 6: Figure S4 for RCC and NSCLC). Notably, the dose-level E-R did not appear to be consistent with the overall E-R. This result appeared more evident in the E-R for melanoma, where the by-dose E-R curves for 1, 3, and 10 mg/kg are approximately parallel. This pattern of E-R suggested that nivolumab clearance may be associated with the probability of OR, such that patients with inherently lower clearance tend to respond better. Clearance was a highly significant predictor of response, and the relationship between clearance and probability of OR was consistent across patients at all dose levels (Fig. 4). Although the results of TGD modeling were different for each tumor type, there were no apparent relationships between tumor shrinkage rates and exposure. Tumor progression rate decreased with increasing exposure for melanoma, NSCLC, and RCC; however, higher exposures appear to be required for NSCLC to achieve the maximum decrease in tumor progression rate. The dose of ≥ 3 mg/kg Q2W provided the maximum decrease in tumor progression rate across all tumor types (Fig. 5).

Overall these results showed that nivolumab is well tolerated up to 10 mg/kg Q2W. Moreover, D-R/E-R relationships for efficacy suggested that nivolumab at 1 mg/kg Q2W may be active for high-immunogenic tumor types of melanoma and RCC. However, a dose of 3 mg/kg Q2W may be required for the less-immunogenic tumor type of NSCLC. Based on these findings, the dose of nivolumab at 3 mg/kg Q2W was selected as a monotherapy dose across tumor types.

## Discussion

By targeting the patient’s immune system, I-O therapies such as nivolumab provide the potential to identify a uniform dose and schedule across multiple tumor types. In this study, we integrated clinical efficacy, safety, RO, and D-R/E-R modeling from a large phase Ib trial to select a nivolumab monotherapy dose for further evaluation. The totality of the clinical, pharmacodynamic (RO), and D-R/E-R evidence indicated that a nivolumab dosing regimen of 3 mg/kg Q2W would be likely to maximize efficacy without risking the emergence of safety and tolerability signals. Therefore, this dosing regimen was selected for evaluation in subsequent phase III trials.

Peripheral pharmacodynamic markers such as PD-1 RO did not provide meaningful demarcation for dose selection; because peripheral RO was saturated at relatively low exposures that corresponded to a nivolumab dose of to 0.3 mg/kg Q2W. The utility of peripheral RO data is also hampered by our limited understanding of the relationships between peripheral RO and intra-tumoral RO and immune-modulating activity in the tumor microenvironment. Interestingly, nivolumab treatment resulted in minor increases in activated T cells in peripheral blood with no evidence of D-R (data not shown). The peripheral pharmacodynamic markers have shown limited D-R relationship and may not be used to select a dose for cancer immunotherapy agents. Newer holistic approaches, such as systems pharmacology modeling, may provide a better understanding of the association between peripheral markers and efficacy that could be used in drug development plans.

Results from the investigation of exposure-efficacy relationships for various efficacy endpoints indicate that tumor types such as melanoma and RCC reached plateaus in derived efficacy at lower exposures compared with NSCLC. These relationships were seen for other measures of efficacy, such as response rate, which represents an early clinical endpoint, and TGD, which is less impacted by duration of follow-up or unconventional responses seen with cancer immunotherapy.

During the E-R analyses, it was noted that responders appeared to have higher nivolumab exposures than non-responders at the same dose level. To further investigate this observation, E-R analysis was performed at each dose level. The predicted probability lines for each dose level (1 vs 3 vs 10 mg/kg) were in parallel; thus, the E-R of nivolumab may be confounded by nivolumab clearance. Interestingly, after a visual observation of higher concentration for responders within a dose level, an E-R was also observed within each dose even if the exposure for non-responders was higher compared with exposure of non-responders at lower dose levels. This indicates that nivolumab baseline clearance, in addition to exposure, may be a predictor for efficacy.

The baseline clearance of nivolumab was lower for complete and partial responders compared with patients with stable or progressive disease. This relationship between nivolumab baseline clearance and efficacy appears to be independent of the established relationship between clearance and nivolumab exposure. Notably, clearance was higher in patients with low baseline serum albumin, which has been reported to be a risk factor for poor prognosis in patients with cancer [18]. In addition, for monoclonal antibodies for cancer therapy, drug clearance may be a surrogate for tumor-related factors such as tumor burden and disease status that are not fully accounted for by patient-specific covariates such as LDH or Eastern Cooperative Oncology Group (ECOG) status [19]. In order to differentiate the potential interplay between nivolumab exposure and clearance on efficacy, ongoing studies are refining the nivolumab E-R analyses with larger phase III datasets by including clearance as a covariate in the E-R modeling. However, it is important that contribution of effect of clearance and true E-R should be teased out during dose selection for monoclonal antibodies in cancer drug development. It is also recommended to evaluate a minimum of two dose levels to study the confounding effect of clearance in dose-selection activity.

It should be noted that these analyses were performed irrespective of PD-L1 expression due to the limited availability of these data early in the nivolumab development program. Therefore, the selected dose is likely to provide optimal efficacy in a mixed PD-L1 expression population. Subsequent phase III trials demonstrated that the dose chosen from the integrated evaluation of multiple endpoints across high- and low-immunogenic tumor types resulted in survival benefits to patients irrespective of PD-L1 expression levels [4, 13, 20, 21]. In NSCLC, 1-, 2-, and 3-year OS rates at 3 mg/kg were 56, 42, and 27 %, respectively [20]. Similarly, 1- 2-, 3-, and 4-year OS rates in melanoma at 3 mg/kg were 65, 47, 41, and 35 %, respectively [4]. Further validation of the dose-selection methodology has subsequently been demonstrated by the results of randomized phase III trials in melanoma, NSCLC, and RCC, each meeting their respective primary survival endpoints at the selected dose and schedule [9–12].

Based on the above analyses, 3 mg/kg Q2W offers a unified dose that provides optimized efficacy across melanoma, RCC, and NSCLC tumor types. However, additional studies are required to further optimize the dosing regimen and treatment duration of immunotherapeutic agents.

Although survival is the gold standard endpoint for fully establishing efficacy for anti-cancer agents, early tumor shrinkage could offer an appealing surrogate of survival in the context of dose selection. This approach was evaluated for cancer immunotherapy by correlating ipilimumab exposure, tumor shrinkage, and survival [22]. Taken together, dose selection based on early efficacy endpoints and TGD modeling can provide reasonable confidence that a selected dose will result in meaningful survival benefit.

## Conclusions

In summary, an integrated methodology utilizing phase Ib clinical activity, safety, D-R/E-R, and pharmacodynamic data indicated an optimal biologic nivolumab monotherapy dose of 3 mg/kg Q2W for further development across different tumor types, and this dose was confirmed in subsequent phase III trials that demonstrated the OS benefits of nivolumab in patients with melanoma, NSCLC, and RCC. The approaches and methodologies described may be applied to emerging I-O agents where limited survival data are available during the early stages of clinical development.

## Additional files

Additional file 1: Figure S1. Study design. AE = adverse event; cCR = confirmed complete response; cPD = confirmed progressive disease; CR = complete response; PD = progressive disease; PR = partial response; SD = stable disease; uCR = unconfirmed complete response; Q2W = every 2 weeks; Q8W = every 8 weeks. (PDF 860 kb)
Additional file 2: Table S1. Baseline patient characteristics from CA209-003 in all treated patients. (DOCX 17 kb)
Additional file 3: Table S2. Dose information summary across all tumor types. (DOCX 16 kb)
Additional file 4: Figure S2. Actual dose received versus dosing day. MEL = melanoma; NSCLC = non-small cell lung cancer; RCC = renal cell carcinoma. (PDF 933 kb)
Additional file 5: Figure S3. Exposure-response for efficacy of nivolumab by dose level in RCC. (**A**) Probability of OR vs Cminss, overall and by dose. (**B**) Probability of OR vs CL, overall and by dose. CL = clearance; Cminss = steady-state trough concentration; OR = objective response; RCC = renal cell carcinoma. (PDF 697 kb)
Additional file 6: Figure S4. Exposure-response for efficacy of nivolumab by dose level in NSCLC. (**A**) Probability of OR vs Cminss, overall and by dose. (**B**) Probability of OR vs CL, overall and by dose. CL = clearance; Cminss = steady-state trough concentration; NSCLC = non-small cell lung cancer; OR = objective response. (PDF 784 kb)

## Abbreviations

AE: Adverse event
BOR: Best overall response
*BTS*: Baseline tumor size
Cavgss: Steady-state time-averaged concentration
Cmaxss: Steady-state peak concentration
Cmin: Trough concentration
Cminss: Steady-state trough concentration
CR: Complete response
CRC: Colorectal cancer
CTLA-4: Cytotoxic T-lymphocyte antigen–4
DLT: Dose-limiting toxicity
D-R: Dose-response
E-R: Exposure-response
GFR: Glomerular filtration rate
I-O: Immuno-oncology
LDH: Lactate dehydrogenase
mCRPC: Metastatic castration-resistant prostate cancer
MedDRA: Medical Dictionary for Regulatory Activities
MTD: Maximum tolerated dose
NSCLC: Non-small cell lung cancer
OR: Objective response
ORR: Objective response rates
OS: Overall survival
PD: Progressive disease
PD-1: Programmed death-1
PD-L1: PD-1 ligand
PFSR 24: Progression-free survival rate at 24 weeks
PK: Pharmacokinetics
PPK: Population pharmacokinetics
*PR*: Linear tumor progression rate
PR: Partial response
Q2W: Every 2 weeks
Q3W: Every 3 weeks
RCC: Renal cell carcinoma
RECIST: Response Evaluation Criteria In Solid Tumors
RO: Receptor occupancy
*SR*: Tumor shrinkage rate constant
*t*: Time
TGD: Tumor-growth dynamics
*TS*: Tumor size
