# Supplementary figures and images for: Nivolumab dose selection: challenges, opportunities, and lessons learned for cancer immunotherapy
Source: J Immunother Cancer. 2016 Nov 15;4:72. doi: 10.1186/s40425-016-0177-2 (PMC5109842; doi:10.1186/s40425-016-0177-2)

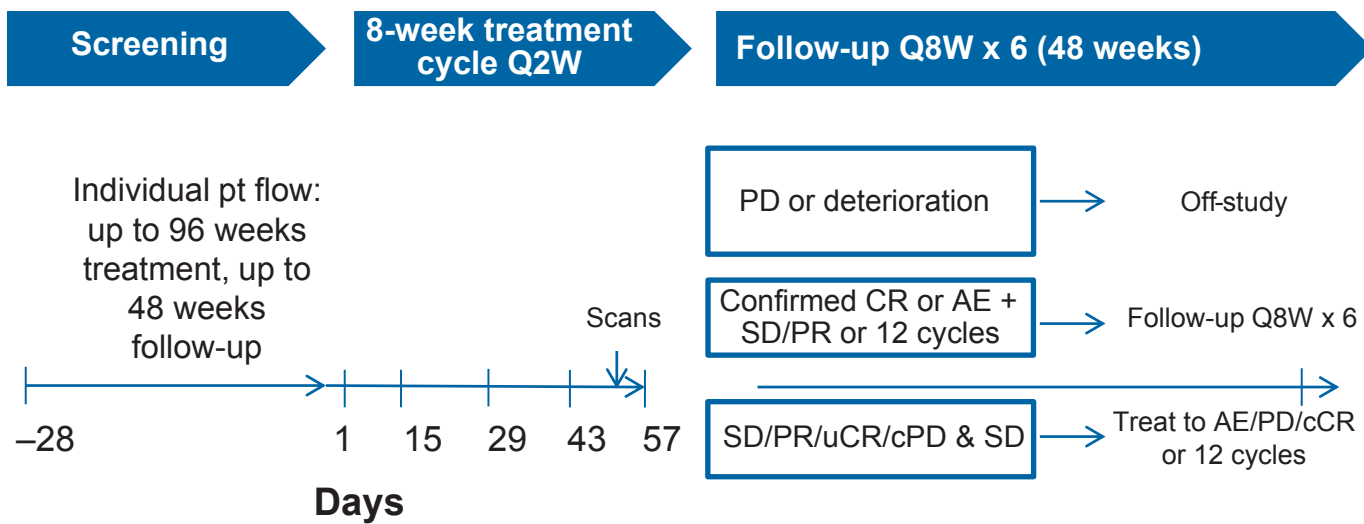

Supplement: Additional file 1: Figure S1. — Study design. AE = adverse event; cCR = confirmed complete response; cPD = confirmed progressive disease; CR = complete response; PD = progressive disease; PR = partial response; SD = stable disease; uCR = unconfirmed complete response; Q2W = every 2 weeks; Q8W = every 8 weeks. (PDF 860 kb) [file 40425_2016_177_MOESM1_ESM.pdf]

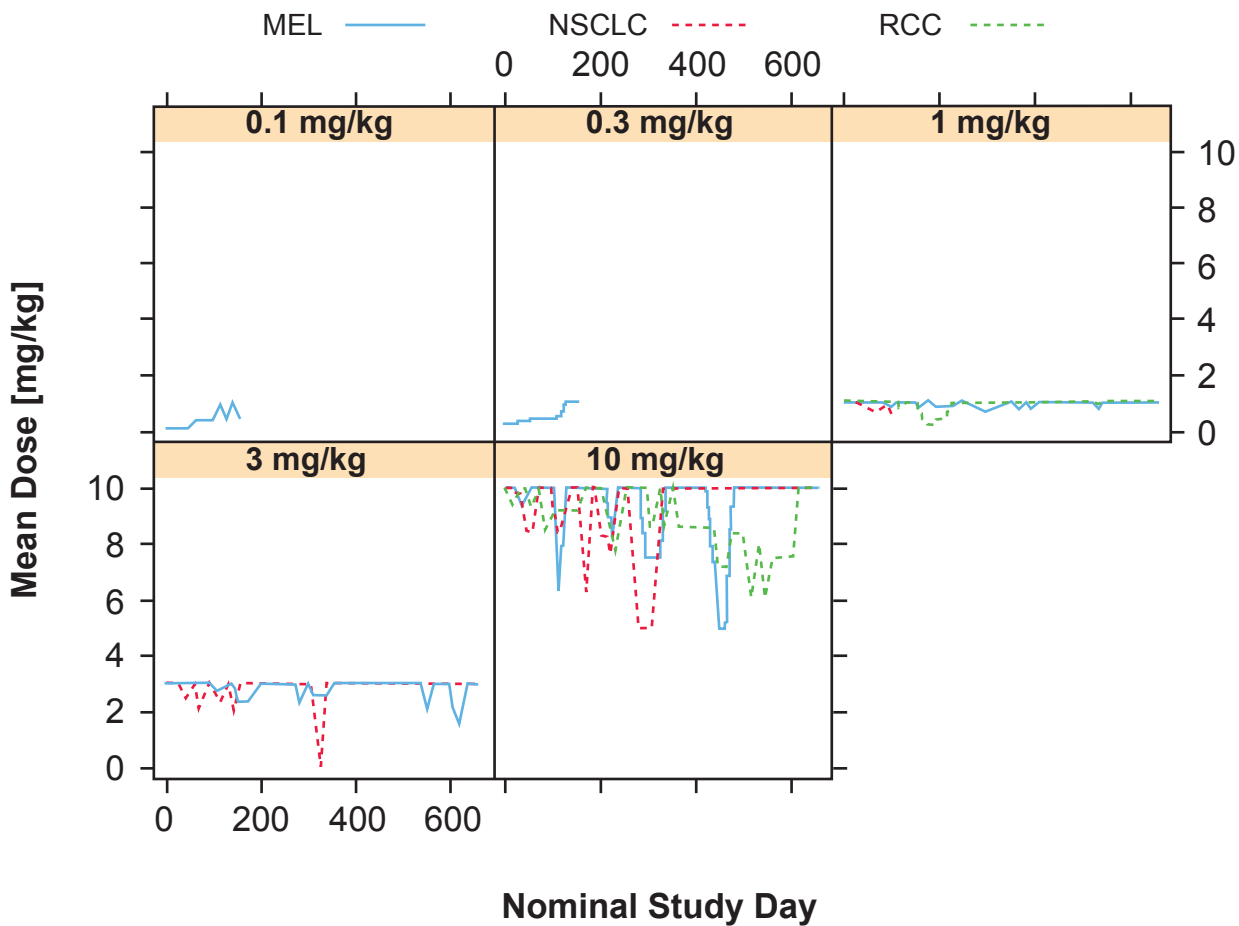

Supplement: Additional file 4: Figure S2. — Actual dose received versus dosing day. MEL = melanoma; NSCLC = non-small cell lung cancer; RCC = renal cell carcinoma. (PDF 933 kb) [file 40425_2016_177_MOESM4_ESM.pdf]

A

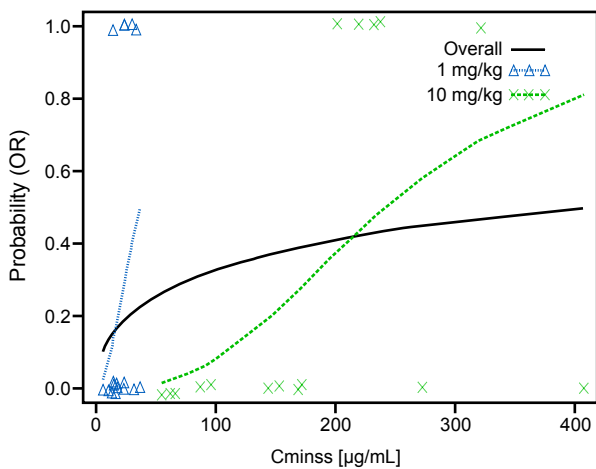

B

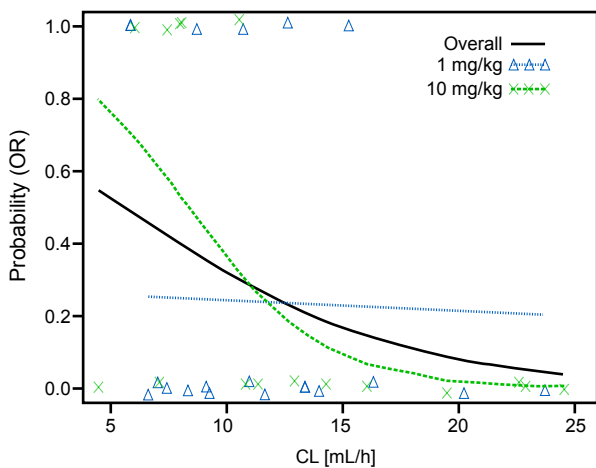

Supplement: Additional file 5: Figure S3. — Exposure-response for efficacy of nivolumab by dose level in RCC. (A) Probability of OR vs Cminss, overall and by dose. (B) Probability of OR vs CL, overall and by dose. CL = clearance; Cminss = steady-state trough concentration; OR = objective response; RCC = renal cell carcinoma. (PDF 697 kb) [file 40425_2016_177_MOESM5_ESM.pdf]

A

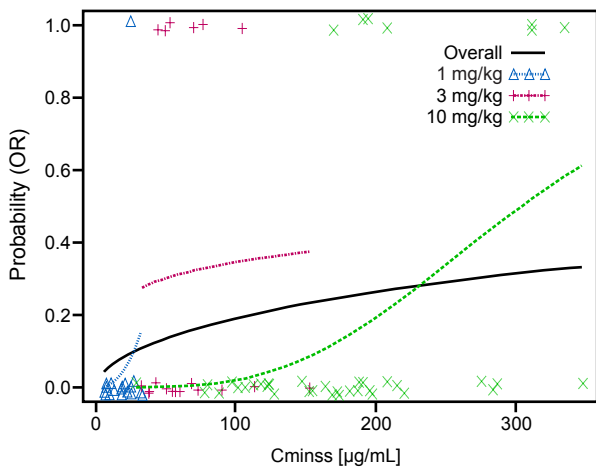

B

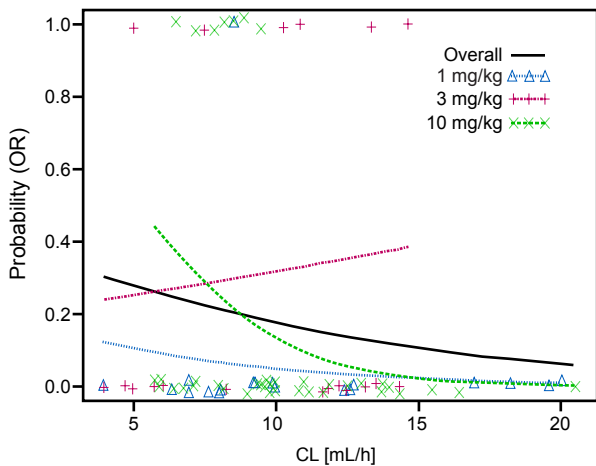

Supplement: Additional file 6: Figure S4. — Exposure-response for efficacy of nivolumab by dose level in NSCLC. (A) Probability of OR vs Cminss, overall and by dose. (B) Probability of OR vs CL, overall and by dose. CL = clearance; Cminss = steady-state trough concentration; NSCLC = non-small cell lung cancer; OR = objective response. (PDF 784 kb) [file 40425_2016_177_MOESM6_ESM.pdf]
