# Supplementary material for: Nivolumab dose selection: challenges, opportunities, and lessons learned for cancer immunotherapy
Source: J Immunother Cancer. 2016 Nov 15;4:72. doi: 10.1186/s40425-016-0177-2 (PMC5109842; doi:10.1186/s40425-016-0177-2)
Supplement: Additional file 2: Table S1. — Baseline patient characteristics from CA209-003 in all treated patients. (DOCX 17 kb) [file 40425_2016_177_MOESM2_ESM.docx]

**Additional file 2: Table S1** Baseline patient characteristics from CA209-003 in all treated patients

| All treated patients (*n* = 306) | |
| --- | --- |
| Median age, years (range) | 63 (29–85) |
| Male, % | 66 |
| Tumor histology, *n* (%) |  |
| MEL | 107 (35) |
| NSCLC | 129 (42) |
| Squamous | 54 (18) |
| Non-squamous | 74 (24) |
| Unknown | 1 (< 1) |
| RCC | 34 (11) |
| mCRPC | 17 (6) |
| CRC | 19 (6) |
| ECOG performance status, *n* (%) |  |
| 0–1 | 300 (98) |
| 2 | 6 (2) |
| Prior therapies, *n* (%) |  |
| 1 | 77 (25) |
| 2 | 85 (28) |
| 3 | 55 (18) |
| ≥ 4 | 88 (29) |

CRC = colorectal cancer; ECOG = Eastern Cooperative Oncology Group; mCRPC = metastatic castration-resistant prostate cancer; MEL = melanoma; NSCLC = non-small cell lung cancer; RCC = renal cell carcinoma.
