# Supplementary material for: Nivolumab dose selection: challenges, opportunities, and lessons learned for cancer immunotherapy
Source: J Immunother Cancer. 2016 Nov 15;4:72. doi: 10.1186/s40425-016-0177-2 (PMC5109842; doi:10.1186/s40425-016-0177-2)
Supplement: Additional file 3: Table S2. — Dose information summary across all tumor types. (DOCX 16 kb) [file 40425_2016_177_MOESM3_ESM.docx]

**Additional file 3: Table S2** Dose information summary across all tumor types

|  | Dose (mg/kg Q2W) | | | | |  |
| --- | --- | --- | --- | --- | --- | --- |
|  | 0.1 (*n* = 17) | 0.3 (*n* = 18) | 1 (*n* = 86) | 3 (*n* = 54) | 10 (*n* = 131) | Total (*N* = 306) |
| Duration of therapy (weeks)^a^ | 24.0 (8–87) | 17.5 (4–90) | 20.0 (2–100) | 22.6 (2–101) | 15.9 (2–122) | 16.1 (2–122) |
| Number of infusions | 11.0 (4–43) | 8.5 (2–43) | 10.0 (1–49) | 9.5 (1–48) | 8.0 (1–51) | 8.0 (1–51) |
| Cumulative dose per patient^b^ | 2.3 (0–36) | 5.4 (1–29) | 10.1 (1–48) | 28.6 (3–138) | 77.8 (2–508) | 30.9 (0–508) |
| Dose intensity per patient (mg/kg/2 weeks)^c^ | 0.1 (0–1) | 0.4 (0–1) | 1.0 (1–1) | 2.9 (2–3) | 9.8 (2–11) | 2.9 (0–11) |

Values are provided as median (range).

^a“^Duration of therapy (weeks)” is defined as the days between the first and last dose + 14 days divided by 7.

^b^“Cumulative dose” is defined as the sum of all doses (in milligrams per kilogram) received by the patient.

^c“^Dose intensity (mg/kg/2 weeks)” is defined per patient as the cumulative dose received divided by the duration of therapy calculated as the number of 2-week dosing intervals.

Q2W = every 2 weeks.
